# An Unusual Musculoaponeurotic Slip of the Brachioradialis as a Potential Cause of Radial Nerve and Radial Recurrent Artery Compression

**DOI:** 10.7759/cureus.103146

**Published:** 2026-02-07

**Authors:** Maria Piagkou, Christos Koutserimpas, Alexandros Samolis, George Triantafyllou, George Tsakotos, Konstantinos Natsis, Georgi P Georgiev

**Affiliations:** 1 Department of Anatomy, School of Medicine, National and Kapodistrian University of Athens, Athens, GRC; 2 Department of Orthopaedics, School of Health Rehabilitation Sciences, University of Patras, Athens, GRC; 3 Department of Anatomy, School of Medicine, Aristotle University of Thessaloniki, Thessaloniki, GRC; 4 Department of Orthopaedics and Traumatology, University Hospital Queen Giovanna - ISUL, Sofia, BGR

**Keywords:** bicipital tendon, brachioradialis muscle, compression, dissection, musculoaponeurotic slip, radial nerve, radial recurrent artery, radial tunnel syndrome, surgical anatomy, variation

## Abstract

Muscular variations in the distal arm and proximal forearm can significantly alter neurovascular relationships and predispose individuals to compressive neuropathies. This dissection report documents a previously unreported musculofascial variant with potential clinical and surgical relevance. During the routine dissection of the right distal arm of a 70-year-old male cadaver, an accessory musculoaponeurotic slip was identified. The structure originated from the deep surface of the brachioradialis (BR) and coursed medially to insert into the bicipital tendon (BT) of the biceps brachii (BB), just proximal to the radial tuberosity. It formed a well-defined arch overlying both the radial nerve (RN) and radial recurrent artery (RRA). The RN exhibited focal indentation and medial displacement at the crossing point, while the RRA was also partially compressed. No other anomalies were noted. This musculoaponeurotic variant differs from previously reported configurations involving the BR and BB, including the accessory brachioradialis muscle (aBR). The simultaneous compression of the RN and RRA by such a structure has not been previously described. Given its location in the operative field of anterior surgical approaches to the elbow, this variation has implications for both diagnosis and surgical planning. This case expands the spectrum of recognized morphological variants that may contribute to radial neuropathy. Awareness of such configurations is essential for clinicians evaluating unexplained RN symptoms and for surgeons performing anterior approaches to the elbow.

## Introduction

Variations in the distal arm and proximal forearm musculature carry important clinical implications, as even minor deviations from typical anatomy can significantly alter local neurovascular relationships. Numerous anatomical investigations have documented muscular abnormalities capable of compressing the radial nerve (RN). These include accessory muscle bellies or aberrant origins of the brachioradialis (ABR) [1], high-origin fibrous slips forming osseomuscular canals [2], split distal brachioradialis (BR) tendons (BRTs) that may entrap the superficial radial nerve (SRN) [3], and accessory brachialis (aB) muscles that cross the RN before merging with the BR [4]. Such variants can create fibromuscular tunnels or constrictive bands, potentially resulting in radial neuropathy, high RN palsy, or symptoms mimicking radial tunnel syndrome [5,6].

Although a broad range of anomalies involving the brachialis-brachioradialis (B-BR) complex has been described, most reported variants involve duplicated muscle bellies, atypical origins, or distal tendon modifications, rather than a direct muscular connection between these two muscles [1,2,7].

A notable exception is the accessory brachioradialis muscle (aBR), which has been observed inserting into the radial tuberosity alongside the biceps brachii (BB) tendon. In a cadaveric study of 176 upper limbs, Rodríguez-Niedenführ et al. [8] reported two cases (1.14%) in which the aBR coursed medially and fused with the bicipital tendon (BT), with the RN passing deep to the structure - highlighting a potential site of neurovascular compression. The RN is the most susceptible in cases of such variations, which typically course between the brachialis (B) and BR muscles before dividing into its deep radial nerve (DRN) and SRN branches [9,10].

The present dissection report describes a rare morphological variant in which a musculoaponeurotic slip arises from the BR, courses medially, and inserts into the BT, forming an arch that crosses over both the RN and the radial recurrent artery (RRA). This report aims to characterize the morphology of this variant, contextualize it within known anatomical patterns, and discuss its potential clinical significance, particularly in relation to radial neuropathy and surgical risk in the distal arm.

## Case presentation

During routine dissection of the right distal arm of a 70-year-old male cadaver, a previously unreported accessory musculoaponeurotic slip was identified. This structure originated from the deep surface of the BR and coursed medially and inferiorly to fuse with the BT of the BB, just proximal to its insertion on the radial tuberosity.

As the slip traversed the interval between the BR and BB, it formed a distinct musculoaponeurotic arch that overlaid both the RN and the RRA. At this crossing, the RN exhibited focal indentation and medial displacement, findings consistent with mechanical compression. The RRA was also partially overlapped and displaced, suggesting concomitant vascular involvement.

**Figure 1:**
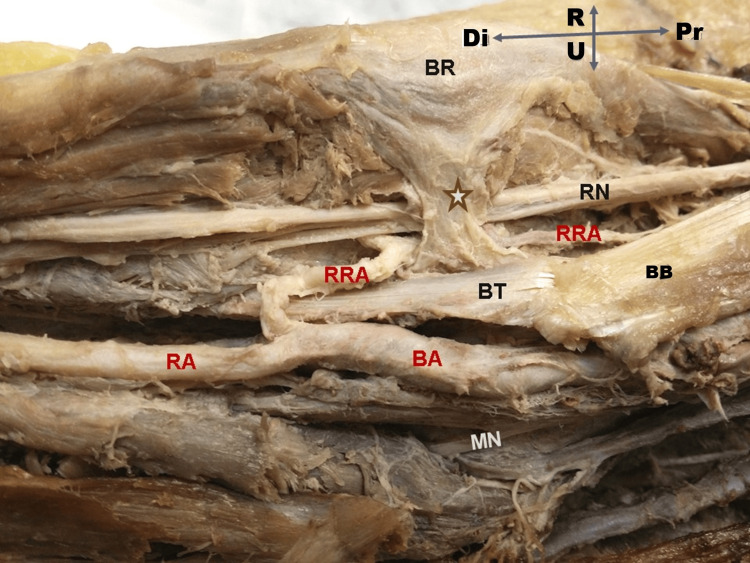
Right forearm dissection Dissection of the right distal arm showing an anomalous musculoaponeurotic slip (★) arising from the deep surface of the brachioradialis (BR) and inserting into the bicipital tendon (BT) of the biceps brachii (BB). The slip forms an arch over the radial nerve (RN), which shows a focal indentation at the crossing point. The radial recurrent artery (RRA) also passes beneath the arch and appears displaced. The brachial artery (BA), radial artery (RA), and median nerve (MN) follow their typical anatomical courses. Orientation: Pr - proximal; Di - distal; R - right; U - ulnar.

No additional muscular, tendinous, or fascial anomalies were observed. Both the BR and BB exhibited typical origins, courses, and insertions. The anomalous slip consisted of a proximal muscular portion, continuous with the BR, transitioning into a distal tendinous segment at its junction with the BT. This configuration supports classification as a true musculoaponeurotic slip, rather than a passive fascial band (Figure 1).

## Discussion

The RN is particularly susceptible to compression as it transitions from the anterior arm to the posterior forearm, especially within the anatomical corridor between the BB, B, and BR - a well-established zone of entrapment. Radial neuropathies at this level have been linked to a range of structural causes, including hypertrophied musculature, trauma, fibrous bands, and muscular anomalies [5-9].

In the present case, a distinct musculoaponeurotic slip arose from the deep surface of the BR and inserted into the BT of the BB. This slip formed a defined arch that crossed both the RN and the RRA, producing focal indentation and medial displacement of the RN and apparent displacement of the RRA. This configuration suggests a previously undocumented mechanism for simultaneous neurovascular compression at the distal arm, with potential clinical implications.

Previous studies have described aBR bellies coursing parallel to the parent muscle but not inserting into the BT [1,2,10]. Likewise, split distal BRTs, as reported by Surendran et al. [3], have been implicated in compression of the SRN, rather than the RN proper. The aB described by Pai et al. [4] crosses the RN en route to merge with the BR, but the directionality and insertion pattern differ from the present case, and no associated vascular displacement was noted.

A particularly relevant comparative variant is the aBR, as described by Rodríguez-Niedenführ et al. [8]. In their cadaveric study, two specimens exhibited a muscle slip arising from the BR and inserting onto the BT, with the RN passing deep to the structure - highlighting its potential for neurovascular entrapment. However, their report did not describe vascular involvement, nor did it characterize the structure as a musculoaponeurotic arch.

The clinical significance of the BR-B interval is well established. Konjengbam and Elangbam [10] identified it as a primary entrapment site in the radial tunnel, while Fuss and Wurzl [11] highlighted the role of fibrous expansions in altering the RN's course. Tonse et al. [12] emphasized the contribution of intermuscular connections in the anterior brachium to RN compression. The present case adds to this body of evidence by demonstrating that such an intermuscular connection can exert dual compression on both the nerve and an accompanying artery.

Modern imaging studies confirm that subtle anatomical variants in the BR, such as fibrous thickenings or anomalous tendon insertions, can compromise RN function [12-15]. Compression near the humeral origin of the BR has been linked with high RN palsy [13], and ultrasonographic (US) studies now identify this region as a key site for potential RN entrapment [16-20].

The anomaly described herein is notable for three reasons: (i) Origin: the slip originates from the BR rather than the B; (ii) Insertion: it fuses with the BT, unlike typical variants that involve the distal BRT or merge with the B; and (iii) Mechanism: it forms a tight, arching musculoaponeurotic loop with potential for simultaneous neural and vascular compression.

Clinically, RN compression proximal to its bifurcation may mimic high RN palsy or radial tunnel syndrome, presenting with weakness of wrist and finger extensors, and dorsolateral forearm paresthesias [6,16]. Displacement of the RRA may further affect collateral circulation around the elbow, with implications during trauma or surgical intervention. From a surgical standpoint, this slip lies within the field of the anterolateral (Henry-type) approach, which accesses the elbow through the BR-B interval. An unrecognized variant may obscure normal tissue planes and increase the risk of iatrogenic injury to the RN or RRA during deep dissection.

This case highlights the importance of detailed anatomical knowledge and careful intraoperative exploration in regions prone to neurovascular variation. A key strength of the present study lies in the meticulous dissection and clear morphological differentiation of the slip from adjacent structures. The photographic documentation and correlation with existing literature further enhance its reproducibility and educational value.

Limitations

Several limitations of this study should be acknowledged. First, the morphological variant was identified in a single cadaveric specimen, which precludes estimation of its prevalence or population variability. Second, due to the unilateral dissection of an isolated limb, laterality symmetry could not be assessed, and it remains unclear whether the variation was bilateral or unilateral. Third, the absence of clinical history limits any correlation between the anatomical findings and potential premortem neurological or vascular symptoms.

Additionally, no histological analysis was performed to examine the microstructure of the slip or the RN at the site of compression. Biomechanical testing was also not conducted, which might have provided insight into the dynamic behavior of the musculoaponeurotic arch during limb movement or muscle contraction. Finally, it is important to note that cadaveric tissues lack physiological muscle tone and vascular pulsatility, potentially underrepresenting the true degree of neurovascular compression that could occur in vivo.

Future directions

Further investigation is needed to determine the prevalence, morphologic variability, and developmental basis of such musculoaponeurotic slips. Larger cadaveric studies, supported by high-resolution imaging modalities such as ultrasound or MRI, may help identify these structures in living subjects and assess their clinical relevance. Histological and biomechanical studies could provide additional clarity on the nature of these slips and their potential role in RN or vascular compression syndromes. Clinically, enhanced awareness of such morphological variations may aid in the evaluation of unexplained proximal radial neuropathies, guide preoperative planning, and improve outcomes in cases of failed decompression or atypical neurovascular presentations.

## Conclusions

We report a previously undocumented musculoaponeurotic variant characterized by a slip arising from the BR deep surface, coursing medially to fuse with the BT. This structure formed an arch over both the RN and the RRA, resulting in focal neural indentation and vascular displacement. This finding expands the known morphological variability of the BB-BR complex and carries important implications for clinical practice. Awareness of this variant is essential for anatomists, surgeons, and radiologists, particularly in the context of unexplained proximal radial neuropathy or during anterior surgical approaches to the elbow. Recognition of such anomalies may improve diagnostic accuracy and reduce the risk of iatrogenic injury in the operating room.
